# Reimagining adolescent well-being through yoga as a public health paradigm

**DOI:** 10.3389/fpsyt.2025.1691915

**Published:** 2026-01-05

**Authors:** Anithamol Babu, Akhil P. Joseph, Jacob Bose, Kalpana Sarathy

**Affiliations:** 1 Marian College Kuttikkanam Autonomous, Kuttikkanam, India; 2 Tata Institute of Social Sciences, Guwahati, India

**Keywords:** adolescent mental health, yoga in schools, public health intervention, neurodiversity inclusion, low- and middle-income countries (LMICs), public health

The escalating prevalence of adolescent mental health disorders—including anxiety, depression, and behavioural dysregulation—demands a reorientation of school health priorities, particularly in low- and middle-income countries (LMICs), where psychiatric services are often inaccessible (1–3). Conventional clinic-based models cannot meet the complexity of these needs (4). What is required are interventions that are low-cost, scalable, culturally resonant, and developmentally appropriate (1–4). Yoga, combining postures, breath regulation, and meditative awareness, has emerged as a promising solution. Its significance lies not in romanticised tradition but in its potential to reduce stress, improve emotion regulation, and build resilience in everyday educational contexts. With one in seven adolescents globally experiencing a mental health condition (5), embedding yoga into school health strategies offers an opportunity for population-level early intervention in communities where stigma and resource constraints limit access to therapy (6–13).

Historically, yoga has been associated not only with spiritual growth but also with holistic health and well-being. Classical texts such as the *Yoga Sutras of Patanjali* emphasised mental clarity and emotional regulation as integral to human flourishing. In the twentieth century, figures such as Swami Kuvalayananda in India initiated scientific studies on the physiological and psychological effects of yoga, which helped integrate it into health and education. Early programs, including the introduction of yoga in Indian schools in the 1970s, highlighted its use for improving concentration and discipline among young people. These historical precedents illustrate that yoga’s educational application is not novel but rather a continuation of longstanding traditions that align bodily practices with psychological resilience.

Yoga’s role is conceptualised within a biopsychosocial–ecological framework that views adolescent well-being as the dynamic interaction of biological, psychological, social, and systemic factors. **Figure 1** illustrates this model by depicting the interconnected layers. The figure evolved through an iterative process: initially informed by Bronfenbrenner’s ecological systems theory (1979) to establish contextual layers; then expanded using Engel’s biopsychosocial model (1977) to foreground mind–body processes; and later refined through principles from the Ottawa Charter for Health Promotion, emphasising enabling environments and participatory approaches (14–16). This evolution transformed a basic ecological diagram into a cohesive multi-level representation of how yoga engages biological regulation, psychological adaptability, social connectedness, and broader systemic influences. Methodologically, the framework synthesises these theoretical strands with empirical insights from school-based intervention studies. At the biological level, yoga supports autonomic functioning and parasympathetic regulation; psychologically, it cultivates mindfulness and self-regulation; socially, it strengthens connectedness and empathy; and ecologically, it shapes classroom climate and school culture. Together, these elements highlight yoga’s potential as a multidimensional practice supporting diverse aspects of adolescent development.

**Figure 1:**
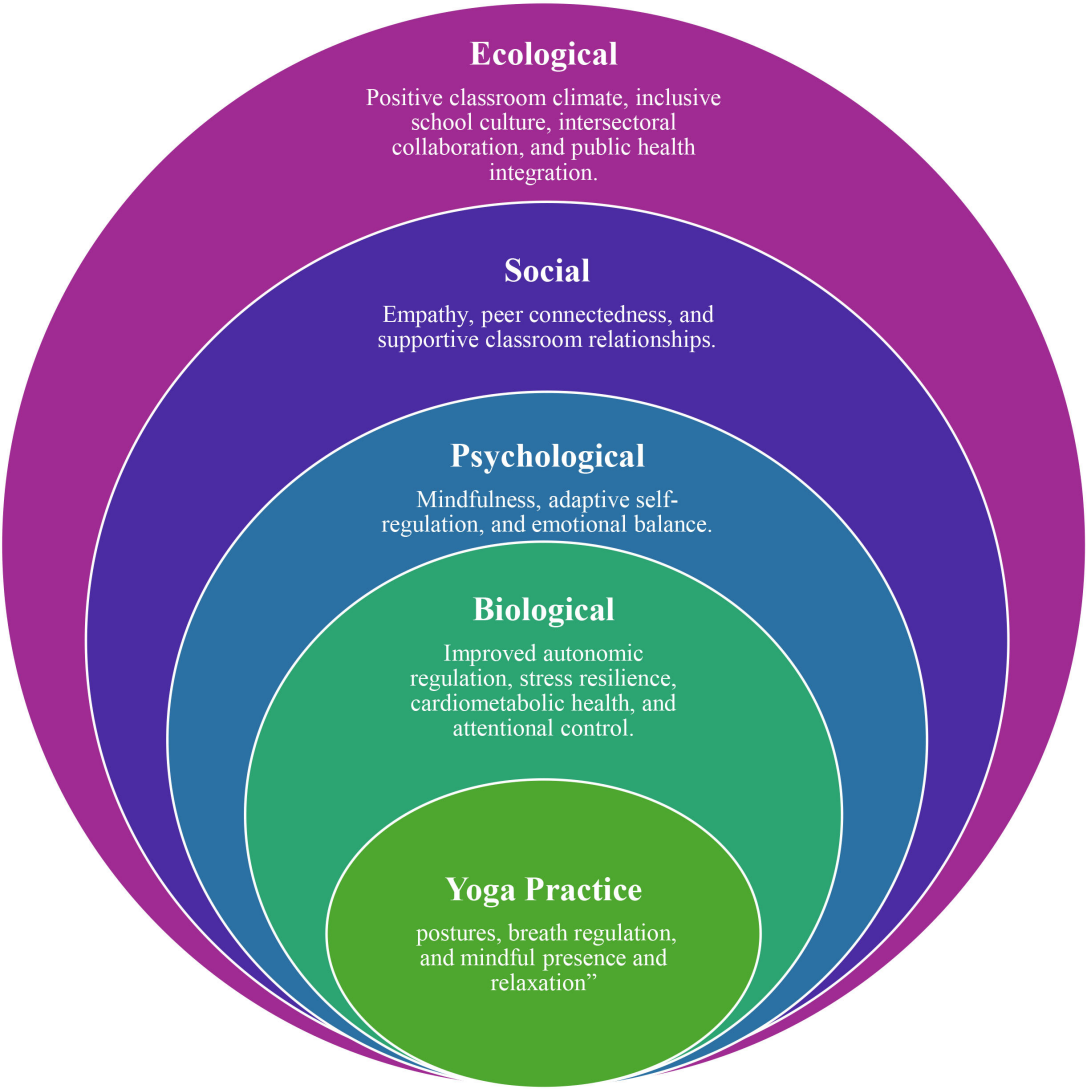
Yoga and Adolescent Well-Being within a Biopsychosocial–Ecological Framework.

Empirical evidence remains encouraging yet methodologically uneven. Across the review of reviews in **Table 1**—each of which applied explicit inclusion criteria encompassing (a) include studies with participants aged ≤18 years from PubMed database, (b) yoga as the primary intervention, (c) Reviews on RCT or controlled experimental design, (d) mental health outcomes such as anxiety, depression, stress, resilience, attention, or metabolic indicators, (e) English-language publications, and (f) school- or community-based settings—school-based and adolescents-focused yoga interventions [17 repeated RCTs across reviews are listed in **Table 2** (6, 17–32] consistently demonstrated reductions in stress, anxiety, and depressive symptoms, alongside improvements in attention, resilience, and coping, positioning yoga as a low-cost, scalable preventive tool within school health systems (33–43). However, the evidence base remains constrained by small samples, heterogeneous intervention designs, short-duration programmes, and limited generalizability across contexts (33–35). Moreover, yoga often exhibits similar benefits to general physical activity in healthy populations, with distinct advantages most evident among adolescents with elevated symptoms (44). While sports, arts-based programs, and social-emotional learning initiatives have also demonstrated promise as scalable and low-cost strategies to promote adolescent well-being, yoga offers distinctive contributions. Team sports can strengthen cooperation, discipline, and physical health, but may not explicitly cultivate attentional control or stress regulation. Arts-based interventions enhance creativity and emotional expression yet often require specialised facilitators and resources. Yoga combines physical activity with mindfulness and breath regulation, bridging somatic, cognitive, and emotional domains. In LMIC contexts where resources are constrained, its minimal equipment requirements and cultural resonance position yoga as a complement rather than a competitor to these established approaches. For yoga to be credibly positioned as a public health intervention, it must be tested comparatively against cognitive-behavioural therapy, structured social-emotional learning curricula, and multi-sport programs to establish whether it provides unique or effective benefits.

**Table 1:** Table of review of reviews.

Author(s), year	Country	Article type	Sample size	Tools/measures	Major results	Limitations	Database
Pandey et al., 2025 (8)	USA	Systematic Review	16 studies	Anxiety scales	Nearly all studies indicated reduced anxiety after yoga intervention	Variable study designs, outcome measures, generalizability	PubMed
Uebelacker et al., 2023 (9)	USA	Systematic Review	27 studies	Anxiety/depression scales	70% showed improvement in anxiety/depression; 58% both, 25% anxiety only	Weak-moderate quality, varied interventions	PubMed
Hagen et al., 2024 (10)	Sweden	Integrative Review	16 studies	Mental health Behaviour & attention, Cognition, Well-being & resilience, etc.	Yoga reduced anxiety, depression, ADHD symptoms, and improved self-control, sleep, cognitive functioning, relaxation, and well-being. High acceptability across settings.	Heterogeneous protocols; several small or uncontrolled studies; methodological inconsistency; limited long-term evidence.	PubMed
Khalsa and Butzer 20216 (11)	USA	Systematic Review of RCTs	39 studies	Mental health (anxiety, depression, stress), Behaviour & self-regulation, Cognition, Physiological indicators, etc.	Most RCTs reported improvements in mental health, emotional regulation, cognition, mindfulness, and physiological stress markers. School-based trials showed the strongest gains.	Wide variability in intervention design; many small samples; inconsistent follow-up; heterogeneous measures; uneven methodological quality	PubMed
Serwacki and Cook-Cottone, 2012 (12)	UK	Scoping Review	40+ studies	Mental health, cognition, resilience, etc.	Yoga improved anxiety, self-concept, resilience, well-being, cognition in most studies	Few neurodiverse studies, heterogeneity	PubMed
Khunti et al., 2022	UK	Systematic Review	21 studies	Stress, mental health scales	Most trials showed reduced stress; yoga feasible as preventive/therapeutic in schools	Small samples, heterogeneity, sparse intervention details	PubMed
Zoogman et al., 2019 (13)	China	Systematic Review/Meta-analysis	38 RCTs (15,730)	Resilience, mental health scales	School-based interventions significantly enhanced resilience in children/adolescents	Considerable heterogeneity, risk of bias in many studies	PubMed
Fulambarkar et al., 2022	USA	Meta-analysis	9 studies (5046)	Stress, depression, anxiety scales	MBIs improved stress (not depression/anxiety); effect significant vs. inactive, not active controls	Small number of studies, effect sizes small, heterogeneity	PubMed
Bronfenbrenner 2079, (14)	USA/India	Systematic Review	47 publications	Mental, emotional, behavioral health	Yoga in schools is viable and potentially efficacious for child/adolescent health	Small samples, weak designs, variability in interventions	PubMed
Engel 1977, Engel 1977, (15)	Sweden	Systematic Review/Meta-analysis	31 articles (30 interventions)	Resilience, well-being, anxiety, etc.	Physical activity (incl. yoga) improved resilience, well-being, anxiety in children/adolescents	Heterogeneity, publication bias, unclear control activities	PubMed
World Health Organization 1986, (16)	Australia	Systematic Review/Meta-analysis	57 trials (meta: 49)	Anxiety, depression, internalizing/externalizing	Universal resilience interventions reduced depression, internalizing, externalizing, distress	Variability in interventions, study quality, bias	PubMed
Khalsa et al., 2012 (17)	China	Systematic Review/Network Meta-analysis	9 RCTs (955)	Depression scales	Yoga most effective among mind-body therapies for adolescent depression	Small sample size, limited certainty, need more studies	PubMed

**Table 2:** Review of RCTs.

Author(s), year	Country	Article type	Sample size (N)	Tools / measures	Major results	Limitations	Database
Noggle et al., 2012 (18)	USA	Randomised Controlled Trial	N = 121 (Yoga = 74; PE = 47)	BASC-2; PSS; Resilience Scale; POMS subscales (tension, anger, fatigue)	Improvements in *anger control* and *fatigue/inertia*; good feasibility; modest effects on general stress	No long-term follow-up; self-report outcomes; underpowered for depression/anxiety	PubMed; PsycINFO
Velásquez et al., 2015 (19)	USA	Randomised Controlled Trial	N = 51–52 (Yoga = 36; PE = 15)	POMS-SF; PANAS-C; PSS; RS; STAXI-2; CAMM	Significant reductions in *tension–anxiety* and *negative affect*; small or no changes in stress or resilience	Small sample; imbalance between groups; PE as active comparator	PubMed; PsycINFO
Mendelson et al., 2010 (20)	Colombia	Randomised Controlled Trial	N = 125 (Yoga = 68; Control = 57)	SDQ (emotional problems, conduct, hyperactivity, peer relations)	Reduced *depressive symptoms*, *aggression*, and *emotional/behavioural difficulties*, especially in younger boys	No teacher ratings; short follow-up; potential self-report bias	PubMed; Scopus
Haden et al., 2014 (21)	USA	Randomised Controlled Trial	N = 97 (Yoga = 51; Control = 46)	SMFQ; Emotion Profile (EP); PIML; RSQ; qualitative focus groups	Improved *stress responses* and *emotional regulation*; modest depressive-symptom improvement	No active comparator; baseline imbalances; limited generalisability	PubMed; PsycINFO
Halliwell et al., 2019 (22)	USA	Randomised Controlled Trial	N = 30 (Yoga = 15; PE = 15)	PANAS-C; CBCL; SPPC; WEMWBS	No significant differences between yoga and PE; both groups improved in mood and well-being	Very small sample; limited dose; possible contamination from PE	PubMed; PsycINFO
Halliwell et al., 2018	UK	Randomised Controlled Trial	N = 344 (Girls only)	Body Esteem Scale; Body Appreciation Scale; PANAS-C	Both groups improved in body image and mood; yoga not superior to PE	PE is a strong active comparator; minimal yoga exposure; short duration	PubMed; Web of Science
White, 2012 (23)	USA	Randomised Controlled Trial	N = 155 (Yoga = 70; WL = 85)	Feel Bad Scale; Coping Strategies; SPPC; self-regulation scales	No significant improvements; yoga group reported *higher perceived stress* (likely increased awareness)	High self-report bias; limited fidelity reporting; analytical limitations	PsycINFO; ProQuest
Case-Smith et al., 2010 (24)	USA	Mixed-Methods / Uncontrolled Trial	N = 21	Child self-concept scales; teacher observations; qualitative thematic analysis	Improved *self-concept*, *focus*, *self-control*; teacher-reported behavioural gains	No control group; small sample; potential expectancy effects	CINAHL; PsycINFO
Conboy et al., 2013 (25)	USA	Quasi-Experimental (with qualitative component)	N = 72 (Yoga = 47; PE = 25)	Self-reported mood, stress, coping, self-regulation; interviews	Reported improvements in *mood*, *stress*, *coping*, and academic/social functioning	No standardised measures; lack of statistical comparison; qualitative-heavy	ERIC; PsycINFO
Klatt et al., 2013 (26)	USA	Uncontrolled Trial	N = 41	ADHD index; behavioural observations; teacher qualitative reports	Reductions in *disruptive behaviours* and *inattention*; improved classroom engagement	No control group; teacher-rated outcomes; weak causal inference	CINAHL; PsycINFO
Ribeiro et al., 2022 (4)	USA	Randomised Controlled Trial	N = 159 (TLS = 80; Control = 79)	PANAS-C; Attitudes Toward Violence; RSQ; school records (attendance, detentions)	Reduced *absences* and *detentions*; improved *school engagement*; moderate emotion-regulation gains	Mixed fidelity; mood improvements modest; limited long-term tracking	PubMed; Scopus
Ribeiro et al., 2022 (4)	USA	Quasi-experimental	N= 49 high-risk adolescents	Stress, psychological distress, coping, affect scales	Reduced anxiety, depression, and global distress; improved coping and emotional regulation.	Non-randomised; high-risk sample; limited generalisability.	PsycINFO, PubMed, CINAHL
Frank et al., 2014 (27)	USA	Two-arm intervention study (Special school—ASD)	N=48	Behavioural checklists, adaptive functioning scales	Reduced irritability, hyperactivity, and social withdrawal (teacher-rated).	No significant parent-rated changes; small sample; short intervention.	PubMed, CINAHL, PsycINFO
Koenig et al., 2012 (28)	India	School-based yoga intervention	N= 76 (ADHD)	Attention, behaviour, ADHD symptom scales	Reduced ADHD symptoms, improved attention and classroom behaviour.	Non-randomised; limited control conditions; short duration.	PubMed, CINAHL
Mehta et al., 2012 (29)	India	Clinical single-arm trial	N= 9 (ADHD)	ADHD symptom scales, clinician severity ratings	Significant reduction in ADHD symptoms during hospital stay.	Very small sample; no control group; short retention.	PubMed, PsycINFO
Blom et al., 2017	USA	Mixed-methods clinical trial	N= 26	Anxiety/depression scales, mindfulness scales, insomnia index; qualitative interviews	Reduced anxiety, depression, insomnia; improved psychological flexibility and well-being.	Small sample; no active control; clinical sample limits generalisability.	PubMed, PsycINFO
Hariprasad et al., 2013 (30)	USA	RCT (School-based)	N= 88	Anxiety/depression scales; stress measures	Reductions in anxiety and depression (not significant vs. controls).	Modest effects; short program; group-level randomisation issues.	PubMed, CINAHL

Thus, yoga should be embedded within a tiered school mental health model, complementing rather than replacing established interventions. Teacher training and peer leadership programs should prioritise trauma-informed facilitation and developmental appropriateness. Curricular content must be adapted to cultural and linguistic contexts through participatory design involving students, educators, and caregivers, rather than imposed as standardised packages. Monitoring and evaluation must combine psychometric tools with ecological indicators such as classroom behaviour, school attendance, and peer relationships, while avoiding exclusive reliance on Western measurement frameworks. Above all, ethical safeguards must guarantee voluntariness, secular framing, and cultural sensitivity, while ensuring that neurodiverse students and children with disabilities are not marginalised. In addition, two implementation safeguards are essential: (a) the introduction of simple contraindication screening and physical-safety protocols to identify students for whom certain postures or breathing techniques may pose risks; and (b) clear opt-in/opt-out participation pathways to respect religious, ethical, and cultural diversity within school communities. These safeguards should be embedded into school-level operational guidelines, ensuring that teachers receive basic training to identify musculoskeletal vulnerabilities, respiratory conditions, and sensory sensitivities that may require modified or alternative practices. Equally important is the establishment of transparent communication mechanisms—such as informed participation forms, parental briefing notes, and culturally sensitive programme descriptions—that enable families to make autonomous decisions without fear of stigma or exclusion.

Therefore, the issue of differentiated learners becomes central. Adolescents are not a homogeneous population; classrooms contain students who learn at varying rates (fast, moderate, and slow learners), as well as those with sensory sensitivities, attention regulation difficulties, or social communication differences. Yoga programs must therefore incorporate flexible pedagogical designs. For fast learners, advanced postures or leadership roles can sustain engagement, while for slower learners, sequences should be broken into stepwise, scaffolded instructions with repeated practice. Importantly, yoga instruction engages multiple learning modalities simultaneously. Kinesthetic learners benefit from physical postures and movement; visual learners are supported through demonstration, diagrams, and visual sequencing, while auditory learners gain from guided breathing instructions and verbal cues. This multimodal structure makes yoga uniquely adaptable to diverse classroom needs, as it allows students to internalise practices through multiple channels of engagement, enhancing both accessibility and retention. Supporting neurodiverse learners requires structured adaptations, including simplified postures, enhanced visual supports, and sensory-sensitive environments with features such as dimmed lighting and reduced noise. These adaptations enable participation without marginalisation and signal that inclusivity is a design principle rather than an afterthought. Encouragingly, evidence from sensory-sensitive and co-designed yoga adaptations demonstrates that such approaches can enhance engagement and reduce overload, reinforcing that inclusivity is a foundational design principle rather than an afterthought (45).

Implementation models from LMIC schools provide useful insights. For example, Anusuya et al. conducted an RCT on yoga-based relaxation techniques, reporting improvements in classroom attentiveness and a reduction in anxiety (46). In Sri Lanka, a six-month yoga-based intervention delivered to Grade 8 students in a post-conflict region—comprising slow-breathing techniques, Surya Namaskāram, and mindfulness —yielded significant reductions in both internalising and externalising behavioural symptoms, as well as reported qualitative improvements in school achievement, family dynamics, and individual health (47). These examples demonstrate that yoga can be implemented sustainably when adapted to a cultural context, integrated into existing school timetables, and supported through teacher training rather than relying on external specialists.

Integration into the school curriculum can also draw lessons from existing physical activity programs that have been successfully embedded into daily routines. For instance, structured movement breaks are commonly introduced in classrooms to improve attention and behaviour, particularly among students with ADHD. Similarly, physical education classes have long been used to promote health, teamwork, and resilience. Yoga can be embedded into these models by incorporating short practices at the beginning of the school day, using brief sequences during transitions, or integrating modules into physical education curricula. These practical applications demonstrate that yoga can complement existing strategies, offering additional benefits of breath regulation, mindfulness, and emotion regulation within familiar school structures.

Cultural reflexivity remains equally important. The institutionalisation of yoga in schools risks reducing an indigenous practice into a depoliticised stress-management technique, while reproducing caste, class, and gender exclusions. When presented as a universal “Eastern” tradition, yoga risks being appropriated into global health discourses in ways that erase its contested histories (48). To avoid cultural tokenism and epistemic erasure, its implementation must embrace plural ontologies of well-being, centre marginalised voices, and resist homogenising knowledge systems. Reflexive implementation is particularly critical in LMICs, where social inequities shape access to both education and health. The path forward lies in combining evidence, inclusivity, and reflexivity. Yoga offers a culturally acceptable, embodied entry point for adolescents in contexts where stigma and resource scarcity constrain mental health engagement. However, the integration must be deliberate: supported by intersectoral collaboration between health and education ministries, embedded into teacher training institutes, financed through sustainable partnerships, and evaluated independently by research institutions. Future studies must move beyond short-term efficacy to include comparative effectiveness trials, cost–benefit analyses in resource-constrained systems, gender-sensitive delivery models, and longitudinal tracking of outcomes such as school retention, academic achievement, and violence prevention.

Thus, evidence indicates that yoga’s benefits are modest yet meaningful. These findings underscore the importance of translating empirical insights into policy frameworks that are scalable, inclusive, and culturally grounded. Future research must rigorously evaluate long-term outcomes, sustainability, and implementation fidelity, ensuring that structured, symptom-focused interventions are led by skilled facilitators who are responsive to contextual needs. To overlook yoga as a transient wellness trend would be shortsighted, for its embodied and culturally legible nature uniquely positions it to bridge the gap between clinical mental health paradigms and everyday practices of emotional regulation within LMIC school settings. The real challenge, then, is not whether yoga should be part of adolescent health policy, but how it can be implemented without reproducing inequities or erasing its cultural complexity. If anchored within a biopsychosocial–ecological framework, co-designed with diverse learners and neurodivergent youth, and embedded in tiered school mental health systems, yoga can serve as a pragmatic and justice-oriented entry point into broader psychosocial support. Its value lies not in replacing established interventions but in expanding the terrain of what is possible—normalising care practices within schools, fostering resilience in contexts of scarcity, and opening space for culturally resonant approaches to adolescent well-being. In this light, yoga should not be perceived as either a universal remedy or a negligible trend; rather, it represents an emerging pedagogical and public health experiment, the outcomes of which will depend on whether it is pursued with reflexivity, inclusivity, and a sustained commitment to evidence-informed practice.

## References

[1] Galagali PM Brooks MJ . Psychological care in low-resource settings for adolescents. Clin Child Psychol Psychiatry. (2020) 25:698. doi: 10.1177/1359104520929741, PMID: 32567351 PMC9137117

[2] Khan F Shehzad RK Chaudhry HR . Child and adolescent mental health services in Pakistan: current situation, future directions and possible solutions. Int Psychiatry. (2008) 5(4):86. doi: 10.1192/s1749367600002253, PMID: 31507958 PMC6734852

[3] Khombo S Khombo K Stoddart RS Sifelani I Sibanda T . Knowledge, attitudes, and uptake of mental health services by secondary school students in Gweru, Zimbabwe. Front Psychol. (2023) 14:1002948. doi: 10.3389/fpsyg.2023.1002948, PMID: 36818083 PMC9930152

[4] Ribeiro WS Grande AJ Hoffmann MS Ziebold C McDaid D Fry A . A systematic review of evidence-based interventions for child and adolescent mental health problems in low- and middle-income countries. Compr Psychiatry. (2022) 121:152358. doi: 10.1016/j.comppsych.2022.152358, PMID: 36508775

[5] World Health Organization . Mental health of adolescents (Fact sheet) (2025). Available online at: https://www.who.int/news-room/fact-sheets/detail/adolescent-mental-health (Accessed November 3, 2025).

[6] Frank JL Kohler K Peal A Bose B . Effectiveness of a school-based yoga program on adolescent mental health and school performance: Findings from a randomized controlled trial. Mindfulness. (2017) 8:544–53. doi: 10.1007/s12671-016-0628-3

[7] Pandey M Dwivedi K Behera N . Effectiveness of yoga and physical exercises on emotional and behavioral problems and academic performance among Indian adolescents: A randomized trial. J Emotional Behav Disord. (2024), 10634266241301372. doi: 10.1177/10634266241301371

[8] Pandey M Dwivedi K Behera N . Self-esteem mediates the effect of yoga-induced mindfulness on adolescents’ emotional and behavioral problems and pro-social behavior. Youth Soc. (2025). doi: 10.1177/0044118x241313065

[9] Uebelacker LA Wolff JC Guo J Conte K Tremont G Kraines M . Assessing feasibility and acceptability of yoga and group CBT for adolescents with depression: A pilot randomized clinical trial. Clin Child Psychol Psychiatry. (2023) 28:525–40. doi: 10.1177/13591045221092885, PMID: 35608457 PMC11927086

[10] Hagen I Nayar SU . Yoga for children and young people’s mental health and well-being: research review and reflections on the mental health potentials of yoga(2014). Available online at: https://www.scienceopen.com/document_file/3fe91bcc-2683-48d2-94c6-cb798facb622/PubMedCentral/3fe91bcc-2683-48d2-94c6-cb798facb622.pdf (Accessed November 3, 2025)., PMID: 10.3389/fpsyt.2014.00035 PMC3980104 24765080

[11] Khalsa S Butzer B . Yoga in school settings: a research review. Ann New York Acad Sci. (2016), 1373. doi: 10.1111/nyas.13025, PMID: 26919395

[12] Serwacki M Cook-Cottone C . Yoga in the schools: a systematic review of the literature. Int J yoga Ther. (2012) 22:101–9. doi: 10.17761/ijyt.22.1.7716244t75u4l702, PMID: 23070680

[13] Zoogman S Goldberg SB Vousoura E Diamond MC Miller L . Effect of yoga-based interventions for anxiety symptoms: A meta-analysis of randomized controlled trials. Spirituality Clin Pract. (2019) 6:256–78. doi: 10.1037/scp0000202

[14] Bronfenbrenner U . The Ecology of Human Development: Experiments by Nature and Design. Harvard University Press (1979).

[15] Engel GL . The need for a new medical model: A challenge for biomedicine. Science. (1977) 196:129–36. doi: 10.1126/science.847460, PMID: 847460

[16] World Health Organization . Ottawa charter for health promotion (1986). WHO Regional Office for Europe. Available online at: https://www.who.int/publications/i/item/ottawa-charter-for-health-promotion (Accessed November 3, 2025).

[17] Khalsa SBS Hickey-Schultz L Cohen D Steiner N Cope S . Evaluation of the mental health benefits of yoga in a secondary school: a preliminary randomized controlled trial. J Behav Health Serv Res. (2012) 39:80–90. doi: 10.1007/s11414-011-9249-8, PMID: 21647811

[18] Noggle JJ Steiner NJ Minami T Khalsa SBS . Benefits of yoga for psychosocial well-being in a US high school curriculum: a preliminary randomized controlled trial: A preliminary randomized controlled trial. J Dev Behav Pediatrics: JDBP. (2012) 33:193–201. doi: 10.1097/DBP.0b013e31824afdc4, PMID: 22343481

[19] Velásquez AM López MA Quiñonez N Paba DP . Yoga for the prevention of depression, anxiety, and aggression and the promotion of socio-emotional competencies in school-aged children. Educ Res Evaluation: Int J Theory Pract. (2015) 21:407–21. doi: 10.1080/13803611.2015.1111804

[20] Mendelson T Greenberg MT Dariotis JK Gould LF Rhoades BL Leaf PJ . Feasibility and preliminary outcomes of a school-based mindfulness intervention for urban youth. J Abnormal Child Psychol. (2010) 38:985–94. doi: 10.1007/s10802-010-9418-x, PMID: 20440550

[21] Haden SC Daly L Hagins M . A randomised controlled trial comparing the impact of yoga and physical education on the emotional and behavioural functioning of middle school children: Original Article. Focus Altern Complementary Therapies. (2014) 19:148–55. doi: 10.1111/fct.12130, PMID: 25147479 PMC4136379

[22] Halliwell E Dawson K Burkey S . A randomized experimental evaluation of a yoga-based body image intervention. Body Image. (2019) 28:119–27. doi: 10.1016/j.bodyim.2018.12.005, PMID: 30660059

[23] White LS . Reducing stress in school-age girls through mindful yoga. J Pediatr Health Care: Off Publ Natl Assoc Pediatr Nurse Associates Practitioners. (2012) 26:45–56. doi: 10.1016/j.pedhc.2011.01.002, PMID: 22153143

[24] Case-Smith J Shupe Sines J Klatt M . Perceptions of children who participated in a school-based yoga program. J Occup Ther Schools Early Intervention. (2010) 3:226–38. doi: 10.1080/19411243.2010.520246

[25] Conboy LA Noggle JJ Frey JL Kudesia RS Khalsa SBS . Qualitative evaluation of a high school yoga program: feasibility and perceived benefits. Explore (New York N.Y.). (2013) 9:171–80. doi: 10.1016/j.explore.2013.02.001, PMID: 23643372

[26] Klatt M Harpster K Browne E White S Case-Smith J . Feasibility and preliminary outcomes for Move-Into-Learning: An arts-based mindfulness classroom intervention. J Positive Psychol. (2013) 8:233–41. doi: 10.1080/17439760.2013.779011

[27] Frank JL Bose B Schrobenhauser-Clonan A . Effectiveness of a school-based yoga program on adolescent mental health, stress coping strategies, and attitudes toward violence: Findings from a high-risk sample. J Appl School Psychol. (2014) 30:29–49. doi: 10.1080/15377903.2013.863259

[28] Koenig KP Buckley-Reen A Garg S . Efficacy of the Get Ready to Learn yoga program among children with autism spectrum disorders: a pretest-posttest control group design. Am J Occup Therapy: Off Publ Am Occup Ther Assoc. (2012) 66:538–46. doi: 10.5014/ajot.2012.004390, PMID: 22917120

[29] Mehta S Shah D Shah K Mehta S Mehta N Mehta V . Peer-mediated multimodal intervention program for the treatment of children with ADHD in India: one-year followup. Nternational Scholarly Res Notices. (2012), 419168. doi: 10.5402/2012/419168, PMID: 23316384 PMC3539379

[30] Hariprasad VR Arasappa R Varambally S Srinath S Gangadhar BN . Feasibility and efficacy of yoga as an add-on intervention in attention deficit-hyperactivity disorder: An exploratory study. Indian J Psychiatry 55(Suppl. (2013) 3):S379–84. doi: 10.4103/0019-5545.116317, PMID: 24049203 PMC3768216

[31] Henje Blom E Tymofiyeva O Chesney MA Ho TC Moran P Connolly CG . Feasibility and preliminary efficacy of a novel RDoC-based treatment program for adolescent depression: “training for awareness Resilience and action“ (TARA)-A pilot study. Front Psychiatry. (2016) 7:208. doi: 10.3389/fpsyt.2016.00208, PMID: 28138319 PMC5237634

[32] Bazzano AN Sun Y Chavez-Gray V Akintimehin T Gustat J Barrera D . Effect of yoga and mindfulness intervention on symptoms of anxiety and depression in young adolescents attending middle school: A pragmatic community-based cluster randomized controlled trial in a racially diverse urban setting. Int J Environ Res Public Health. (2022) 19:12076. doi: 10.3390/ijerph191912076, PMID: 36231378 PMC9564597

[33] James-Palmer A Anderson EZ Zucker L Kofman Y Daneault J-F . Yoga as an intervention for the reduction of symptoms of anxiety and depression in children and adolescents: A systematic review. Front Pediatr. (2020) 8:78. doi: 10.3389/fped.2020.00078, PMID: 32232017 PMC7082809

[34] Khunti K Boniface S Norris E De Oliveira CM Shelton N . The effects of yoga on mental health in school-aged children: A Systematic Review and Narrative Synthesis of Randomised Control Trials. Clin Child Psychol Psychiatry. (2023) 28:1217–38. doi: 10.1177/13591045221136016, PMID: 36302735 PMC10280666

[35] Weaver LL Darragh AR . Systematic review of yoga interventions for anxiety reduction among children and adolescents. Am J Occup Therapy: Off Publ Am Occup Ther Assoc. (2015) 69:6906180070p1–9. doi: 10.5014/ajot.2015.020115, PMID: 26565100

[36] Andermo S Hallgren M Nguyen T-T-D Jonsson S Petersen S Friberg M . School-related physical activity interventions and mental health among children: a systematic review and meta-analysis. Sports Med - Open. (2020) 6:25. doi: 10.1186/s40798-020-00254-x, PMID: 32548792 PMC7297899

[37] Cai C Mei Z Wang Z Luo S . School-based interventions for resilience in children and adolescents: a systematic review and meta-analysis of randomized controlled trials. Front Psychiatry. (2025) 16:1594658. doi: 10.3389/fpsyt.2025.1594658, PMID: 40458775 PMC12127306

[38] Dray J Bowman J Campbell E Freund M Wolfenden L Hodder RK . Systematic Review of universal resilience-focused interventions targeting child and Adolescent Mental Health in the School Setting. J Am Acad Child Adolesc Psychiatry. (2017) 56:813–24. doi: 10.1016/j.jaac.2017.07.780, PMID: 28942803

[39] Fulambarkar N Seo B Testerman A Rees M Bausback K Bunge E . Review: Meta-analysis on mindfulness-based interventions for adolescents’ stress, depression, and anxiety in school settings: a cautionary tale. Child Adolesc Ment Health. (2023) 28:307–17. doi: 10.1111/camh.12572, PMID: 35765773

[40] Hart N Fawkner S Niven A Booth JN . Scoping review of yoga in schools: Mental health and cognitive outcomes in both neurotypical and neurodiverse youth populations. Children (Basel Switzerland). (2022) 9:849. doi: 10.3390/children9060849, PMID: 35740786 PMC9222138

[41] Luo S Mei Z Fang G Mu G Zhang X Luo S . Effects of mind-body therapies on depression among adolescents: a systematic review and network meta-analysis. Front Public Health. (2024) 12:1431062. doi: 10.3389/fpubh.2024.1431062, PMID: 39050611 PMC11266190

[42] Kerekes N Söderström A Holmberg C Hedman Ahlström B . Yoga for children and adolescents: A decade-long integrative review on feasibility and efficacy in school-based and psychiatric care interventions. J Psychiatr Res. (2024) 180:489–99. doi: 10.1016/j.jpsychires.2024.11.016, PMID: 39547048

[43] Miller S Mendelson T Lee-Winn A Dyer NL Khalsa SBS . Systematic review of randomized controlled trials testing the effects of yoga with youth. Mindfulness. (2020) 11:1336–53. doi: 10.1007/s12671-019-01230-7

[44] Hendriks T Jong J Cramer H . The effects of yoga on positive mental health among healthy adults: A systematic review and meta-analysis. J Altern Complementary Med. (2017) 23:505. doi: 10.1089/acm.2016.0334, PMID: 28437149

[45] Khin CM . Designing a sensory yoga play kit for children with sensory differences (2022). OCAD University. Available online at: https://openresearch.ocadu.ca/id/eprint/3920/1/Khin_Chan%20Myae_2022_MDES_INCD.pdf (Accessed November 3, 2025).

[46] Anusuya US Mohanty S Saoji AA . Effect of Mind Sound Resonance Technique (MSRT - A yoga-based relaxation technique) on psychological variables and cognition in school children: A randomized controlled trial. Complementary Therapies Med. (2021) 56:102606. doi: 10.1016/j.ctim.2020.102606, PMID: 33197570

[47] Sivashankar JT Surenthirakumaran R Doherty S Sathiakumar N . Implementation of a non-randomized controlled trial of yoga-based intervention to reduce behavioural issues in early adolescent school-going children in Sri Lanka. Globalization Health. (2022) 18:27. doi: 10.1186/s12992-022-00819-3, PMID: 35248094 PMC8898460

[48] McCartney PSD . Spiritual bypass and entanglement in Yogaland: How Neoliberalism, Soft Hindutva and Banal Nationalism Facilitate Yoga Fundamentalism (2019). Available online at: https://www.academia.edu/38580765/Spiritual_bypass_and_entanglement_in_Yogaland_How_Neoliberalism_Soft_Hindutva_and_Banal_Nationalism_Facilitate_Yoga_Fundamentalism (Accessed November 3, 2025).

